# Supplementary material for: Comparison of Post-Vaccination Cellular Immune Response in Patients with Common Variable Immune Deficiency
Source: Vaccines (Basel). 2024 Jul 25;12(8):843. doi: 10.3390/vaccines12080843 (PMC11360582; doi:10.3390/vaccines12080843)
Supplement: Supplementary file 1 [file vaccines-12-00843-s001.zip › vaccines-3068119-supplementary.pdf]

Supplementary material.

Table S1. Percentage and absolute content of lymphocyte subpopulations in the blood in groups of patients with PID vaccinated primarily with one and two doses of the vaccine.

		Period	Values				Between groups
			1 dose (n = 6)		2 doses (n = 9)		
			Med (Q1-Q3)	95 % CI	Med (Q1-Q3)	95 % CI	
Leukocytes	Abs.	Before vaccination	5418 (4768-7178)	[4385; 9116]	5230(4463-6201)	[4380; 6437]	p = 0,66
		After 24 ± 3 days	5962 (4468-6909)	[3971; 7852]	5688(4258-7504)	[3877; 8312]	p = 0,66
		RLMEM[1]	Dose: t (17) = -0,4, p = 0,66, Time: t (13) = -0,2, p = 0,86, Dose × Time: t (13) = 0,01, p = 1,00				
Lymphocytes	%	Before vaccination	33,9(28,7-37,6)	[28,1; 42,7]	33,5(28,9-36,1)	[28,5; 36,4]	p = 0,69
		After 24 ± 3 days	33,1(24,3-36,7)	[22,3; 40,1]	34,9(27,9-40,8)	[24,0; 40,9]	p = 0,28
		RLMEM	Dose: t (21) = -0,4, p = 0,70, Time: t (13) =-1,4, p = 0,17, Dose × Time: t (13) = 1,6, p = 0,14				
	Abs.	Before vaccination	1856(1709-2227)	[1608; 2628]	1638(1263-2017)	[1232; 2129]	p = 0,46
		After 24 ± 3 days	1937(1419-2231)	[887; 2630]	1649(1145-2729)	[1115; 3028]	p = 0,86
		RLMEM	Dose: t (21) = -0,8, p = 0,46, Time: t (13) = -0,6, p = 0,58, Dose × Time: t (13) = 0,6, p = 0,57				
CD3+CD19–	%	Before vaccination	78,3(73,4-83,5)	[73,3; 91,4]	86,7(78,0-90,5)	[77,8; 90,9]	p = 0,17
		After 24 ± 3 days	82,8(75,6-87,7)	[74,3; 92,3]	86,1(78,5-88,5)	[77,9; 88,6]	p = 0,70
		Analysis of changes	**p < 0,001**		p = 0,11		–
		RLMEM	Dose: t (13) = 1,4, p = 0,20, Time: t (13) = 4,0, p = 0,001, Dose × Time: t (13) = -4,4, p < 0,001				
CD3+CD19–	Abs.	Before vaccination	1475(1356-1716)	[1300; 2089]	1472(996-1803)	[963; 1856]	p = 0,67
		After 24 ± 3 days	1615(1101-1852)	[765; 2108]	1200(962,5-2142)	[942; 2359]	p = 0,80
		RLMEM	Dose: t (19) = -0,4, p = 0,67, Time: t (13) =-0,4, p = 0,70, Dose × Time: t (13) = 0,2, p = 0,83				
CD	%	Before vaccination	9,8(6,7-14,3)	[2,5; 15,6]	5(3,0-13,0)	[3,0; 13,0]	p = 0,28

		After 24 ± 3 days	7,5(5,1-11,0)	[2,8; 14,0]	6,4(2,8-12,2)	[2,2; 12,2]	p = 0,60
		Analysis of changes	**p = 0,009**		p = 0,53		–
		RLMEM	Dose: t (13) = -1,1, p = 0,30, Time: t (13) = -2,1, p = 0,06, Dose × Time: t (13) = 2,1, p = 0,05				
	Abs.	Before vaccination	221(109-291)	[44; 292]	82(45-198)	[40; 212]	p = 0,21
		After 24 ± 3 days	171(53-218)	[51; 223]	79(36-274)	[35; 297]	p = 0,57
		RLMEM	Dose: t (14) = -1,2, p = 0,23, Time: t (13) = -1,6, p = 0,13, Dose × Time: t (13) = 1,5, p = 0,15				
CD3+CD4+	%	Before vaccination	35,5(21,6-41,6)	[19,8; 41,8]	33,6(27-43,4)	[26,8; 44,9]	p = 0,62
		After 24 ± 3 days	33,6(22,7-42,6)	[20,5; 44,6]	33(28,6-41,3)	[28,4; 41,9]	p = 0,75
		RLMEM	Dose: t (14) = 0,5, p = 0,63, Time: t (13) = 0,1, p = 0,93, Dose × Time: t (13) = -0,5, p = 0,64				
	Abs.	Before vaccination	687(361,5-848,5)	[357; 1045]	536(429,5-818,5)	[414; 892]	p = 0,73
		After 24 ± 3 days	612,5(456,3-802,5)	[208; 873]	495(410-914,5)	[388; 960]	p = 0,95
		RLMEM	Dose: t (16) = -0,4, p = 0,73, Time: t (13) = -0,8, p = 0,47, Dose × Time: t (13) = 0,7, p = 0,52				
CD3+CD8+	%	Before vaccination	43,9(36,2-49,7)	[33,8; 49,8]	48(37-52,4)	[35,2; 53,3]	p = 0,67
		After 24 ± 3 days	42,4(36-49,1)	[34,5; 51,5]	43,3(37,8-47,9)	[34,5; 48,5]	p = 0,85
		RLMEM	Dose: t (15) = 0,4, p = 0,67, Time: t (13) = -0,2, p = 0,88, Dose × Time: t (13) = -0,5, p = 0,64				
CD3+CD8+	Abs.	Before vaccination	835,5(753-926)	[633; 972]	749(536-945)	[481; 1005]	p = 0,56
		After 24 ± 3 days	794(509-1057)	[383; 1270]	651(548,5-1073)	[527; 1173]	p = 0,75
		RLMEM	Dose: t (21) = -0,6, p = 0,57, Time: t (13) = -0,5, p = 0,63, Dose × Time: t (13) = 0,2, p = 0,81				
CD3–	%	Before vaccination	9,6(7,5-12,3)	[4,0; 13,4]	6,6(4,5-12,2)	[3,9; 13,1]	p = 0,32
		After 24 ± 3 days	9,3(7,1-11,5)	[5,8; 14,0]	6,7(4,6-9,9)	[4,1; 10,3]	p = 0,34

		RLMEM	Dose: t (14) = -1,0, p = 0,34, Time: t (13) = 0,1, p = 0,91, Dose × Time: t (13) = 0,1, p = 0,94				
	Abs.	Before vaccination	209(124-246)	[70,0; 249]	87(81-262)	[79; 342]	p = 0,23
		After 24 ± 3 days	162(100-284)	[66,5; 294]	92(68-234)	[65; 312]	p = 0,31
		RLMEM	Dose: t (17) = -1,2, p = 0,25, Time: t (13) = -0,5, p = 0,66, Dose × Time: t (13) = 0,3, p = 0,80				

[1] – A robust linear mixed effects model (RLMEM) was used, with time since vaccination (“Time”) and one or two vaccine doses (“Dose”) as fixed factors and individual patients as random factors. The number of degrees of freedom is represented as t(df). Post hoc comparisons (between groups at control points and between control points for each group) were made by constructing appropriate contrasts based on the estimated model.
